# Putting the Focus Back on the Patient: How Privacy Concerns Affect Personal Health Information Sharing Intentions

**DOI:** 10.2196/jmir.6877

**Published:** 2017-09-13

**Authors:** Mohamed Abdelhamid, Joana Gaia, G Lawrence Sanders

**Affiliations:** ^1^ College of Business Administration Department of Information Systems California State University Long Beach Long Beach, CA United States; ^2^ Management Science and Systems School of Management University at Buffalo Buffalo, NY United States

**Keywords:** information sharing, health information technology, privacy, personal health information

## Abstract

**Background:**

Health care providers are driven by greater participation and systemic cost savings irrespective of benefits to individual patients derived from sharing Personal Health Information (PHI). Protecting PHI is a critical issue in the sharing of health care information systems; yet, there is very little literature examining the topic of sharing PHI electronically. A good overview of the regulatory, privacy, and societal barriers to sharing PHI can be found in the 2009 Health Information Technology for Economic and Clinical Health Act.

**Objective:**

This study investigated the factors that influence individuals’ intentions to share their PHI electronically with health care providers, creating an understanding of how we can represent a patient’s interests more accurately in sharing settings, instead of treating patients like predetermined subjects. Unlike privacy concern and trust, patient activation is a stable trait that is not subject to change in the short term and, thus, is a useful factor in predicting sharing behavior. We apply the extended privacy model in the health information sharing context and adapt this model to include patient activation and issue involvement to predict individuals’ intentions.

**Methods:**

This was a survey-based study with 1600+ participants using the Health Information National Trends Survey (HINTS) data to validate a model through various statistical techniques. The research method included an assessment of both the measurement and structural models with post hoc analysis.

**Results:**

We find that privacy concern has the most influence on individuals’ intentions to share. Patient activation, issue involvement, and patient-physician relationship are significant predictors of sharing intention. We contribute to theory by introducing patient activation and issue involvement as proxies for personal interest factors in the health care context.

**Conclusions:**

Overall, this study found that although patients are open to sharing their PHI, they still have concerns over the privacy of their PHI during the sharing process. It is paramount to address this factor to increase information flow and identify how patients can assure that their privacy is protected. The outcome of this study is a set of recommendations for motivating the sharing of PHI. The goal of this research is to increase the health profile of the patients by integrating the testing and diagnoses of various doctors across health care providers and, thus, bring patients closer to the physicians.

## Introduction

### Background

This study investigates critical factors that influence individuals’ decisions to share their personal health information (PHI) electronically among health care providers. The paper focuses on individuals’ information privacy concern, patient-physician relationship, trust in health care providers, and health-related factors such as patient activation and issue involvement. There is extensive literature investigating the behavior of people who seek information on the Web, yet there is only a modest amount of research studying what factors influence private health information sharing. The impetus for investigating the barriers to sharing health information was the 2009 Health Information Technology for Economic and Clinical Health (HITECH) Act. The passing of the HITECH Act marked a significant change in the appropriate protections and processes for sharing health information. With the signing of the HITECH Act of 2009, by President Obama, incentives and guidelines were established for health care providers for the use of Health Information Exchanges (HIEs). This Act provides a strong rationale for the development of HIEs. Indeed, it is recognized that exchanging patients’ health information electronically improves the quality of care, reduces medical errors, and reduces medical costs [1-3]. However, patients’ information cannot be shared, unless patients agree to share via an HIE. The value of HIE, therefore, is directly related to the relative ease of sharing among providers, payers, and patients [4]. There is a noticeable effort in literature trying to investigate the reasons affecting the willingness to share. Sharing patients’ health information has been considered an urgency to promote investments in health care information technology [5]. Patients’ decisions not to share may result in medical errors and undesired health outcomes. Our aspiration to understand the psychology behind patients’ decision comes from our desire to address barriers to sharing and enhance motivators of sharing to help patients make better choices for their own health [5]. The findings of this paper will help health care stakeholders and policy makers enhance sharing of PHI to achieve better health care.

The motivation for this study comes from the willingness to provide practical and theoretical implications to the health care field for understanding factors affecting patients’ intentions to share their PHI. Few studies have investigated ways to persuade patients to share their health information [6]; in fact, factors influencing patients’ decisions related to sharing health information have not been studied thoroughly [7], and this study aims to address the gap in existing literature. Once this understanding is achieved, health care providers can promote motivational factors to help improve the sharing of PHI according to patients’ needs and concerns and this will lead to better health outcomes and reduced medical costs for the entire population. In this study, the factors of interest are those that explain the decision to share or not to share PHI with health care providers.

### Prior Literature

The literature that addresses attitudes toward sharing PHI is scarce and often characterized by studies with small sample sizes or only applicable to one group of the patient population [8,9]. Extant literature in attitudes toward sharing PHI usually addresses either the patients’ willingness to share or barriers to sharing PHI.

Patients are typically very willing to share their PHI and have a positive attitude toward sharing practices [10-12], and this willingness is enhanced when potential privacy concerns are addressed [11,13]. Patients who trust their clinicians [14] and who can understand the health benefits brought by sharing PHI practices [6] will also be willing to share their PHI.

The studies that report barriers to sharing PHI point out a variety of factors that hinder participation in HIEs or electronic sharing of PHI. The foremost barrier encountered in the studies is related to the privacy and security of PHI. Patients are apprehensive about who will have access to their PHI [6,8,14], how it will be used in HIEs [15], and the intentions of the PHI users [16]. Patients who have control over how much information they share and who they share it with are more prone to sharing [9,15,17]. Patients who do not have a perception of the benefits brought by PHI sharing are also less willing to share their information [8,18]. Low income, ethnic diversity [14], general health status, certain personality traits [19], and exiting medical conditions (eg, depression) [8] have all been identified as factors that hinder engaging in PHI sharing practices.

In summary, the majority of studies in the attitude toward sharing PHI arena have shown that patients encounter many barriers that thwart the above-mentioned willingness to share PHI. It is therefore critical to understand how consumers can be educated and how their concerns can be addressed to achieve higher sharing rates. Our ultimate goal is to help patients achieve optimal health outcomes while protecting their privacy.

### Theoretical Foundation

This study investigates the impact of concern for information privacy, trust in providers, patient activation, issue involvement and patient-physician relationship on the intention to share PHI. The overall contribution of this study is to provide theoretical and practical insights to address the privacy and trust barriers in consent. Moreover, we use traits such as patient activation to predict patients’ intentions to share their PHI. Unlike trust and privacy, these traits are not subject to changes at least in the short term and, thus, are stable and robust predictors of individuals’ intentions.

The proposed model has been theoretically developed based on the extended privacy calculus model [20]. Many studies in the information systems field focused on understanding people’s intentions and behavior toward information technology, and based their theoretical foundations on the theory of reasoned action and the theory of planned behavior [21,22]. However, the extended privacy calculus model is specifically intended to predict intention of information disclosure in the Internet environment, and thus is more appropriate for our study. The model proposes that an individual’s intention to disclose information in Web-based transactions depends on their privacy concerns, trust in the system, and their personal interest in the context of the transaction.

**Figure 1:**
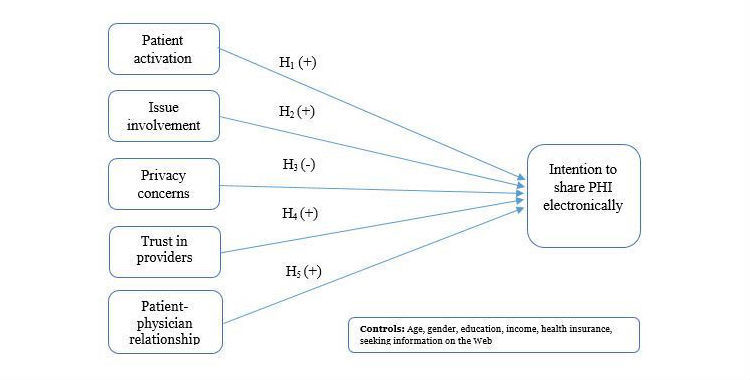
Conceptual research model.

### Research Model and Hypotheses

We adapt the extended privacy calculus model and use it in the context of health care information systems to predict individuals’ intentions to share their PHI electronically among providers. We introduce patient activation and issue involvement predictors of personal interest to engage in the sharing behavior in the health care context, as seen in Figure 1.

### Patient Activation

A person’s decision to engage in a behavior depends on their motivation to engage in that behavior [23,24]. Motivation to engage in a behavior has shown a positive impact on seeking information and sharing information [25]. In the context of health information, health motivators impact an individual’s decisions to seek information [26].

When investigating behavioral intention, many studies adopt theories that focus on the assessment of costs and benefits such as the expectancy theory [27]. However, these factors are unstable traits and therefore measuring them does not offer health care providers constant estimation of their impact on patients’ intentions. In general, patients have a positive attitude about the benefits of HIEs regarding improving health outcomes [15,17,28,29] and health motivators have an influence on people's intentions to engage in behaviors related to health outcomes [30]. Thus we have integrated patient activation into the model.

We define patient activation as the extent to which an individual wants to seek available resources and skills to engage in preventive health [31]. Patients’ health lifestyles are relatively stable traits that provide constant estimation of people’s intentions based on their health lifestyles measures [32]. Thus, patient activation is an appropriate stable predictor of patients’ intentions to share their health information with providers.

H_1_: Patient activation is positively associated with the intention to share PHI electronically with providers.

### Issue Involvement

Sharing PHI is influenced by personal contextual relevance [33], which is captured by issue involvement [6]. We define issue involvement as personal relevance, that is, how relevant a specific health issue is to each patient.

This study investigates the decision to share health information on the Web, and thus an involved person would be someone who is frequently visiting providers, has a number of diseases, and has a severe health status [6]. Issue involvement is defined in the literature as the extent to which an issue is of relevance and importance to the patient [34]. In the context of this study, the issue of concern is health and people’s decisions to disclose health information electronically [6]. People who are more involved in the issue, health in this case, are expected to be more willing to share [33]. Therefore, the degree to which an individual may be contextually involved may influence his or her decision to share information. Thus, based on these results the second hypothesis is as follows:

H_2_: Issue involvement is positively associated with the intention to share PHI electronically.

### Privacy

Privacy is defined as having control over who has access to medical records and being aware that those records are protected. With low control and no awareness of existing safeguards, the patients will have high privacy concerns.

A potential drawback in sharing information in HIE is the risk of patient privacy invasion and information security violations, which are increasing concerns due to the growing amount of health information exchanged electronically. Health care providers are driven by greater participation and systemic cost savings irrespective of benefits to individual patients. HIE is a new technology, and the risk of information breaches and privacy issues are not understood by the patients yet, especially when there is a lack of education. Moreover, when security breaches occur, patients are not compensated for their losses, which makes sharing of high privacy and security a risk. Simon et al [8] investigated the barriers to consent using 62 patients in a focus group and reported that privacy and security concerns, lack of knowledge of possible benefit to an individual’s health, and the need for more information about the consent process are the main factors affecting sharing decisions.

While many people want to share their PHI with preferred providers [15,17], they are concerned with the privacy and security of their information with regard to HIEs.

H_3_: Privacy concern is negatively associated with the intention to share PHI electronically.

### Trust

Trust is defined as the extent to which the patients have confidence in their health care providers. Lack of trust can be associated with other users who misuse or mishandle the information, with the system itself and its ability to protect information, or with people who illegally breach the system and misuse the information. The probability of any of these type or misuse happening increases when information is exposed to more people and shared and exchanged across multiple systems.

The more trust people have in the system the more engaged [35,36] they will be. For patients with HIV, trust in clinicians is associated with positive attitude toward sharing health information [14]. Therefore, a lack of trust is a barrier to patients consenting to share their PHI among providers. Trust has been associated with usability, that is, people will use a system more if they trust it. If patients are uncertain of why or how their information is used or shared, they can develop a lack of trust and thus be less engaging. Allowing patients to control who will access their health information and what information is available for access should lead to more engagement.

H_4_: Trust in providers is positively associated with the intention to share PHI electronically.

### Patient-Physician Relationship

The time physicians spend with their patients is a dimension that defines the strength of the patient-physician relationship [37]. Communication and social factors such as warmth, feeling, help, and understanding are factors shaping a patient-physician relationship [38]. Therefore, a patient-physician relationship can be defined as the mixture of social strength, time spent, understanding, and help that characterizes the connection. When patients consent to share their PHI with providers, they are expecting physicians to use this information to make better decisions [17]. Thus, patients share information because they seek information represented in better doctors’ opinions, diagnoses, and prescriptions. The strength of the patient-physician relationship is associated with patients’ decisions to share information with their physicians [34,39].

H_5_: Patient-physician relationship is positively associated with the intention to share PHI electronically.

## Methods

### Data

A dataset from the National Cancer Institute was used to investigate the proposed hypotheses. The institute conducts the Health Information National Trend Survey known as HINTS, and this study used data collected in 2014 (HINTS 4 Cycle 4). The sample was nationally representative and thus made the findings generalizable.

As reported in the literature review, most studies have limitations for generalizability. The survey asks questions about participants’ health conditions and health lifestyles, intention to share PHI, and a variety of related questions. Data from 1606 participants were used to analyze the conceptual model. Table 1 shows a distribution of participants. Of the total, 38.61% (620/1606) were males and 61.39% (986/1606) were females. The survey targeted adults who were aged 18 years and above. The average age was about 54 years, with a standard deviation of approximately 16. Among the participants, 18.99% (305/1606) have a high school degree, 60.33% (969/1606) have a college degree, and 20.67% (332/1606) have a postgraduate degree.

### Measurement

Education, age, race, income level, health insurance, and the use of the Internet to look for health-related information were used as controls in the model. A formative measure of issue involvement was constructed using three items: the number of chronic diseases, the frequency of doctors’ visits, and health status [6]. The factor loading for each of the items were 0.81, 0.76, and 0.67 as indicated in Multimedia Appendix 1. A composite factor score was calculated for the variable and the new variable was used in structural equation modeling (SEM) as an observed variable. Table 2 shows the results of the factor analysis. Since the factor analysis was conducted based on correlations rather than covariance, the use of different scales for each item was not an issue. The rest of the measures were constructed using reflective items. Intention to share PHI electronically consisted of six items, patient-physician relationship consisted of seven items, patient activation consisted of four items, and privacy concern involved two items. Trust in providers consisted of a single-item variable. Single items were acceptable if the question did not leave room for interpretation [39]. Single-item variables were used in information systems research that used SEM in the health care context [6]. The constructs were validated using confirmatory factor analysis and exploratory factor analysis (EFA). Table 2 shows the items and scales for all independent variables.

### Reliability and Validity

We used STATA version 14.1 (StataCorp LP, College Station) to recode and analyze the data. To assess the validity of our measures, we performed confirmatory factor analyses on all questionnaire items using the STATA 14 SEM tool. The results reported in Table 2 show that all factor loadings for the constructs are substantial and significant. The goodness of fit indices—comparative fit index (CFI), root mean square error of approximation (RMSEA), and standardized root mean square residual (SRMR)—indicated a good fit of the measurements model for both pre- and post-intervention [40]. According to Hu and Bentler [40], the threshold for CFI is >0.90, for RMSEA is <0.06, and for SRMR is <0.10. The threshold for the Tucker-Lewis index (TLI), which is also known as the non-normed fit index, is >0.90. The Cronbach alpha values were all well above the threshold value of point 0.70 [41]. The composite reliability scores were also all well above the threshold of 0.70 [42]. Table 2 shows the Cronbach alpha and composite reliability scores for the measures. Convergent validity was assessed by calculating the average variance extracted (AVE), where each indicator was related to only one construct. The AVE values for all constructs exceeded 0.5, which was the desirable cutoff suggesting a convergent validity [43] (see Table 2). Discriminant validity was established for the study, because the AVE values for any two constructs exceeded the squared construct intercorrelation for each pair [43].

**Table 1:** Sample demographics (N=1606).

Demographic characteristics			Sample size, n (%)
**Gender**			
	Male		620 (38.61)
	Female		986 (61.39)
**Age (in years)**			
	18–25		53 (3.30)
	26–35		200 (12.45)
	36–45		244 (15.19)
	46–55		338 (21.05)
	56–65		395 (24.6)
	Over 65		376 (23.41)
**Income (**US $ **)**			
	Under $10,000		97 (6.04)
	$10,000 to under $15,000		87 (5.42)
	$15,000 to under $20,000		86 (5.35)
	$20,000 to under $35,000		189 (11.77)
	$35,000 to under $50,000		234 (14.57)
	$50,000 to under $75,000		300 (18.68)
	$75,000 to under $100,000		230 (14.32)
	$100,0000 or more		383 (23.85)
**Education**			
	High school or less		305 (18.99)
	Some college (2 years)		509 (31.69)
	College degree (4 years)		460 (28.64)
	Postgraduate degree		332 (20.67)
**Number of chronic diseases**			
	0		489 (30.45)
	1		453 (28.21)
	2		309 (19.24)
	3		220 (13.70)
	4		85 (5.29)
	5		42 (2.62)
	6		8 (0.50)

**Table 2:** Factor analysis, reliability, and validity.

Construct items		CFA^a^ factor loadings	EFA^b^ factor loadings					Cronbach alpha		CR^c^		AVE^d^	
			1	2	3	4	5						
**Patient-physician relationship (PPR)**									.94		0.9		0.67
	PPR1	0.78	0.84	0.01	0.03	0	−0.03						
	PPR2	0.80	0.83	0	0.02	−0.03	0.05						
	PPR3	0.83	0.87	−0.01	−0.01	0.01	−0.02						
	PPR4	0.87	0.89	−0.02	−0.01	−0.01	−0.02						
	PPR5	0.82	0.86	−0.02	−0.03	0.02	−0.03						
	PPR6	0.82	0.85	0.03	0.01	0.01	0.01						
	PPR7	0.79	0.82	0.01	0	0	0.06						
**Intention to share (INT)**									.94		0.9		0.68
	INT1	0.80	−0.02	0.86	−0.02	0	0.02						
	INT2	0.84	0	0.89	−0.03	0.02	0						
	INT3	0.87	−0.03	0.91	−0.02	0	0.02						
	INT4	0.78	0.02	0.83	0.05	−0.01	−0.04						
	INT5	0.85	0.03	0.88	0	0.02	−0.03						
	INT6	0.81	0.01	0.86	0.02	−0.02	0.01						
**Patient activation (PA)**									.92		0.9		0.67
	PA1	0.81	0.03	0	0.88	−0.02	−0.01						
	PA2	0.75	−0.02	0	0.85	−0.04	0.09						
	PA3	0.87	−0.01	0	0.90	0.04	−0.02						
	PA4	0.84	0.01	0	0.89	0.03	−0.06						
**Issue involvement (II)**									.61		N/A		N/A
	II1	N/A	−0.04	0	−0.03	0.81	−0.04						
	II2		−0.05	0.03	0.08	0.67	0.05						
	II3		0.1	−0.03	−0.05	0.76	0.01						
**Privacy concern (PC)**									.75		0.7		0.59
	PC1	0.75	0	−0.04	−0.01	0.01	0.89						
	PC2	0.78	0.03	0.04	0.01	0.01	0.88						
**Overall goodness of fit**														
	RMSEA^e^	0.056											
	CFI^f^	0.966											
	TLI^g^	0.958											
	SRMR^h^	0.025											

^a^CFA: confirmatory factor analysis.

^b^EFA: exploratory factor analysis.

^c^CR: composite reliability.

^d^AVE: average variance extracted.

^e^RMSEA: root mean square error of approximation.

^f^CFI: comparative fit index.

^g^TLI: Tucker-Lewis index.

^h^SRMR: standardized root mean square residual.

Table 3 shows the correlation matrix and the discriminant validity. The factor loadings of EFA showed strong loadings for items that belong to the same construct and very low loadings for items that belong to different constructs. No cross-loadings were observed, thus further establishing discriminant and convergent validity (see Table 2).

### Common Method Variance

Data collected through a common method can suffer from common method variance (CMV), in which the relationship between the constructs is affected by the use of a single method [44]. CMV was assessed through a marker variable technique [45]. A marker variable is a variable that is theoretically unrelated to one or more of the variables measured in the study. *Worrying* was used as a marker variable that is theoretically unrelated to patient activation. The construct variables and the theoretically unrelated variable should have a low correlation. The correlation between the marker variable and health knowledge was 0.004 and not statistically significant, thus meeting the threshold of being below 0.1 [45]. After controlling for the marker variable using the approach introduced by Lindell and Whitney [45], the level of significance and the direction of the correlation between patient activation and every other variable did not change. Therefore, there is no evidence that the data was biased due to CMV.

**Table 3:** Correlation matrix and discriminant validity.

Variable	Average	SD	1	2	3	4	5	6	7	8	9
PPR^a^	1.66	0.68	0.819^e^								
Intention	2.8	0.92	0.045 (*P*=.07)	0.825^e^							
PA^b^	3.59	0.53	−0.043 (*P*=.08)	0.137 (*P*<.001)	0.819^e^						
II^c^	−0.04	0.99	0.077 (*P*<.001)	−0.051 (*P*=.04 *)*	−0.103 (*P*<.001)	N/A					
PC^d^	1.94	0.52	0.261 (*P*<.001)	−0.120 (*P*<.001 *)*	−0.022 (*P*=.37 *)*	0.0077 (*P*=.76 *)*	0.768^e^				
Trust	3.38	0.72	−0.665 (*P*<.001)	−0.024 (*P* 34 *)*	0.047 (*P*=.06 *)*	−0.062 (*P*=.01 *)*	−0.248 (*P*<.001)	N/A			
Seek Internet	0.7	0.46	0.059 (*P*=.02)	0.182 (*P*<.001)	0.079 (*P*=.002 *)*	−0.246 (*P*<.001)	0.056 (*P*=.02 *)*	−0.057 (*P*=.02 *)*	N/A		
Health insurance	0.92	0.27	−0.042 (*P*=.09)	−0.007 (*P*=.79 *)*	0.009 (*P*=.70 *)*	−0.036 (*P*=.15 *)*	−0.033 (*P*=.19 *)*	0.069 (*P*<.001 *)*	0.046 (*P*=.06 *)*	N/A	
Male	0.39	0.49	0.005 (*P*=.84)	−0.029 (*P*=.24 *)*	−0.012 (*P*=.65 *)*	−0.017 (*P*=.50 *)*	0.083 (*P*<.001)	0.045 (*P*=.07 *)*	−0.044 (*P*=.08 *)*	0.065 (*P*=.009)	N/A
Age	53.77	15.8	−0.068 (*P*=.006)	−0.211 (*P*<.001 *)*	−0.094 (*P*<.001)	0.314 (*P*<.001)	0.001 (*P*=.96 *)*	0.092 (*P*<.001)	−0.291 (*P*<.001)	0.106 (*P*<.001)	0.164 (*P*<.001)

^a^PPR: patient-physician relationship.

^b^PA: patient activation.

^c^II: issue involvement.

^d^PC: privacy concern.

^e^Bold numbers in diagonal are the square root of average variance extracted.

## Results

STATA version 14.1 was used to analyze the data. SEM was used to test the hypothesized model. Estimates derived from the SEM analysis were used to test the research hypotheses. The overall goodness of fit statistics of the structural model indicated a good model fit (RMSEA = 0.053, CFI = 0.91, and RMSR=0.068). In the first hypothesis, we proposed a relationship between patient activation and the intention to share PHI electronically. The path coefficient is positive and significant (βHM = 0.102, *P*<.001; see Table 4), suggesting that higher patient activation yields higher intention to share PHI electronically, thus supporting hypothesis 1. In the second hypothesis, we argued that higher issue involvement (II) would yield greater intention to share PHI electronically. The results support the second hypothesis (βII = 0.093, *P*=.001; see Table 4). The third hypothesis states that privacy concern (PC) is negatively associated with the intention to share PHI electronically. The path coefficient is negative and significant (βPC = −0.160, *P*<.001; see Table 4), suggesting that higher privacy concern yields lower intention to share PHI electronically. Thus, hypothesis 3 is supported. In hypothesis 4, we proposed a positive relationship between trust in providers and the intention to share PHI electronically. The trust in provider coefficient is −0.003 and not significant (*P*=.99), indicating that trust in providers does not play a significant role in people’s intentions to share their PHI electronically with the providers. There is no evidence to support hypothesis 4. Hypothesis 5 states that better patient-physician relationship (PPR) will yield higher intention to share PHI electronically. The path coefficient is positive and significant (βPPR = 0.103, *P*=.003; see Table 4), which provides support to hypothesis 5.

**Table 4:** Model results—dependent variable: intention to share personal health information electronically.

Variables		Standardized coefficients	*P* values	95% CI
Issue involvement		0.093	.001	0.036 to 0.149
Patient activation		0.102	<.001	0.051 to 0.153
Privacy concern		−0.160	<.001	−0.224 to −0.095
Patient-physician relationship		0.103	.003	0.034 to 0.171
Trust in providers		−0.003	.99	−1.39 to 1.385
**Controls**					
	Seek Internet information	0.105	<.001	0.054 to 0.157
	Have health insurance	−0.004	.88	−0.054 to 0.046
	Male	0.005	.84	−0.043 to 0.054
	Other controls (age, income, and education)			
**Overall goodness of fit**					
	RMSEA^a^	0.053		
	CFI^b^	0.91		
	TLI^c^	0.90		
	SRMR^d^	0.068		

^a^RMSEA: root mean square error of approximation.

^b^CFI: comparative fit index.

^c^TLI: Tucker-Lewis index.

^d^SRMR: standardized root mean square residual.

**Figure 2:**
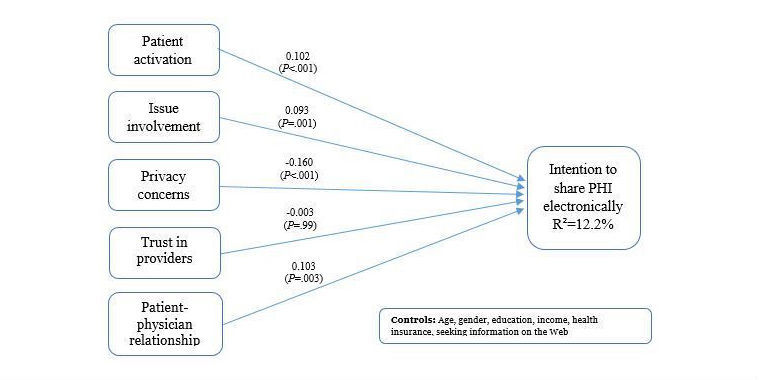
Research model and results.

## Discussion

### Principal Findings

Overall, the results provide empirical support for the core hypotheses. Issue involvement, patient activation, privacy concern, and patient-physician relationship are all important and influenced individuals’ intentions to share their PHI electronically with providers. We do not have data on actual behavior related to sharing PHI electronically with providers, but we suggest that this is an important area for future research. Trust in providers showed no significant relationship with the intention to share PHI. Among all the five main independent variables, privacy concern has the most influence on the intention to share. Standardized coefficients have been reported in Table 4. Standardized coefficient results show that the absolute magnitude for privacy concern is the highest with 0.160. The next highest number being patient-physician relationship is only 64.4% (0.103/0.160) of the magnitude of privacy concern. Given that privacy concern has a negative significant association unlike other significant variables, privacy concern is a major barrier to sharing PHI with providers. This finding provides practical implications to health care providers and policy makers of the significance of this concern. Health care providers and policy makers should prioritize their efforts and focus on addressing individuals’ privacy concerns. In addition, health care providers should invest in educating people on the privacy policies that protect patients’ information and privacy.

Historically, health care providers have focused on educating patients on the benefits of sharing their PHI, by emphasizing both cost and error reductions. Our study shows that there should be a shift in patient education, with a more salient focus on addressing privacy concerns. By making patients more aware of existing privacy policies and security measures in place, the health care providers are creating an environment where the patients are more likely to share their PHI, and therefore still able to achieve cost and error reduction benefits.

Patient-physician relationship has the highest positive significant magnitude. As patient-physician relationship consists of health professionals spending enough time with patients, involving patients in the decision making, helping patients understand steps needed to take care of their own health, and clarifying uncertainty, health professionals should pay particular attention to these factors.

Patient activation is a major factor associated with sharing behavior. It is also an essential element, because unlike trust, patient activation is a trait that is unlikely to change in the short run. Measuring patients’ patient activation will provide outstanding insight into predicting sharing behavior.

Health care providers should focus more on people who are not involved in the issue because higher issue involvements will yield higher intention to share. Individuals who do not participate will be less likely to share, and thus health care providers should focus their attention in making the benefits of sharing more salient to noninvolved patients.

Overall, we propose a shift in education on two separate fronts: on the one hand, patient education is crucial to generate a perceived technological safe environment for sharing PHI electronically, and on another front, we suggest that physician education is as important as patient education. Physicians who are aware of the dimensions of the patient-physician relationship can improve the said relationship, leaving the patient more prone to PHI sharing, achieving better medical decisions, reduction in medical errors, and cost benefits.

### Limitations

This study was based on a dataset that is publicly available. The constructs included in the study were therefore limited to those that are available in the collected survey. Another limitation is that the construct for measuring the trust in providers was based on a single-item variable. Wanous et al [39] state in their study, which specifically evaluates single-item measures, that single-item measures are reliable if there is little room for misunderstanding by the participants. For future studies, the trust in providers construct should be collected using multiple items.

### Conclusions

This research contributes to the extended privacy calculus model. The privacy calculus model was used in this study because it is a very robust way to explain and predict people’s intention to share their PHI. This assertion was substantiated by illustrating that the extended privacy calculus model is viable for explaining information sharing in the health care context.

In particular, this paper integrates personal interest variables in the health care context such as issue involvement and patient activation. The primary takeaway is that the research model provides theoretical and practical implications for sharing health care information. Privacy concerns are a central stage in modern society and the crown jewel is the sharing of PHI, and our model is a substantial first step in understanding the relevant variables related to sharing.

## Appendix

Multimedia Appendix 1 Operationalization of constructs.

## Abbreviations

AVE: average variance extracted
CFA: confirmatory factor analyses
CFI: comparative fit index
CMV: common method variance
CR: composite reliability
EFA: exploratory factor analysis
HIE: health information exchange
HINTS: health information national trend survey
HITECH: health information technology for economic and clinicians health
II: issue involvement
PA: patient activation
PC: privacy concern
PHI: personal health information
PPR: patient-physician relationship
RMSEA: root mean square error of approximation
SEM: structural equation modeling
SRMR: standardized root mean square residual
TLI: Tucker-Lewis index
