# Supplementary material for: Putting the Focus Back on the Patient: How Privacy Concerns Affect Personal Health Information Sharing Intentions
Source: J Med Internet Res. 2017 Sep 13;19(9):e169. doi: 10.2196/jmir.6877 (PMC5617905; doi:10.2196/jmir.6877)
Supplement: Multimedia Appendix 1 [file jmir_v19i9e169_app1.pdf]

**Multimedia Appendix**

Table A. Operationalization of Constructs (source HINTS 2014)

| Construct Items | Question | Scale | Coding |
|---|---|---|---|
| **Intention to Share PHI electronically [Dependent Var.]** | How interested are you in exchanging the following types of medical information with a health care provider electronically? | | |
| INT1 | Lab/test results | 1 to 4 (Very-Not at all) | 1 to 4 (Not at all-Very) REVERSE CODED |
| INT2 | Diagnostic information (e.g., medical illnesses or diseases) | | |
| INT3 | Vital signs (e.g., heart rate, blood pressure, glucose levels, etc.) | | |
| INT4 | Lifestyle behaviors (e.g., physical activity, food intake, sleep patterns, etc.) | | |
| INT5 | Symptoms (e.g., nausea, pain, dizziness, etc.) | | |
| INT6 | Digital images/video (e.g., photos of skin lesions) | | |
| **Patient-Physician Relationship** | The following questions are about your communication with all doctors, nurses, or other health professionals you saw during the past 12 months. How often did they do each of the following: | | |
| PPR1 | Give you the chance to ask all the health-related questions you had? | 1 to 4 (Always-Never) | 1 to 4 (Never-Always) REVERSE CODED |
| PPR2 | Give the attention you needed to your feelings and emotions? | | |
| PPR3 | Involve you in decisions about your health care as much as you wanted? | | |
| PPR4 | Make sure you understood the things you needed to do to take care of your health? | | |
| PPR5 | Explain things in a way you could understand? | | |
| PPR6 | Spend enough time with you? | | |
| PPR7 | Help you deal with feelings of uncertainty about your health or health care? | | |
| **Health Motivation** | How much do you think health behaviors like diet, exercise, and smoking determine whether or not a person will develop each of the following conditions? | | |
| HM1 | Diabetes/High blood sugar | 1 to 4 (A lot-Not at all) | 1 to 4 (Not at all-A lot) REVERSE CODED |
| HM2 | Obesity | | |
| HM3 | Heart disease | | |
| HM4 | High Blood Pressure/Hypertension | | |
| **Issue Involvement** | | | |
| II1 | **[FreqGoProvider]** In the past 12 months, not counting times you went to an emergency room, how many times did you go to a doctor, nurse, or other health professional to get care for yourself? | 0-6 | 0-6 |
| II2 | **[# of chronic diseases]** Diabetes or high blood sugar? | Yes or No | Sum of all yeses |
| | High blood pressure or hypertension? | | |
| | A heart condition such as heart attack, angina, or congestive heart failure? | | |
| | Chronic lung disease, asthma, emphysema, or chronic bronchitis? | | |

| | | | |
|---|---|---|---|
| | Arthritis or rheumatism?<br>Depression or anxiety disorder? | | |
| II3 | **[Health Status]** In general, would you say your health is | 1 to 5 (Excellent – Poor) | 1 to 5 (Excellent – Poor) |
| **Privacy Concern** | | | |
| PC1 | How confident are you that safe guards (including the use of technology) are in place to protect your medical records from being seen by people who aren't permitted to see them? | 1 to 3 (Very confident- Not confident) | 1 to 3 (Very confident- Not confident) |
| | Having safeguards (including the use of technology) in place has to do with the security of your medical records. | | |
| PC2 | How confident are you that you have some say in who is allowed to collect, use, and share your medical information? | | |
| | Having a say in who can collect, use, and share your medical information has to do with the privacy of your records. | | |
| **Trust in Providers** | | | |
| TR1 | In the past 12 months, how often did you feel you could rely on your doctors, nurses, or other health care professionals to take care of your health care needs? | 1 to 4 (Always- Never) | REVERSE CODED |
